# Endophytic actinobacteria of medicinal plants: diversity and bioactivity

**DOI:** 10.1007/s10482-015-0502-7

**Published:** 2015-06-21

**Authors:** Patrycja Golinska, Magdalena Wypij, Gauravi Agarkar, Dnyaneshwar Rathod, Hanna Dahm, Mahendra Rai

**Affiliations:** Department of Microbiology, Nicolaus Copernicus University, 87100 Torun, Poland; Department of Biotechnology, SGB Amravati University, Amravati, 444602 Maharashtra India

**Keywords:** Actinobacteria, Antimicrobial activity, Bioactive compounds, Endophytes, Medicinal plants

## Abstract

Endophytes are the microorganisms that exist inside the plant tissues without having any negative impact on the host plant. Medicinal plants constitute the huge diversity of endophytic actinobacteria of economical importance. These microbes have huge potential to synthesis of numerous novel compounds that can be exploited in pharmaceutical, agricultural and other industries. It is of prime importance to focus the present research on practical utilization of this microbial group in order to find out the solutions to the problems related to health, environment and agriculture. An extensive characterization of diverse population of endophytic actinobacteria associated with medicinal plants can provide a greater insight into the plant-endophyte interactions and evolution of mutualism. In the present review, we have discussed the diversity of endophytic actinobacteria of from medicinal plants their multiple bioactivities.

## Introduction

Many types of microbial population such as bacteria and fungi have been found to be associated with the internal tissues of plant as endophytes. The term endophyte was coined by De Bary (1866), which involves the existence of microorganisms inside the infested plant tissues without having negative effects on host plant (Schulz and Boyle 2006). Almost all the plants have been found to be infested with one or more endophytes (Petrini et al. 1992). The microbes are producers of growth promoting metabolites, insect and pest repellents, antimicrobials against plant pathogens, protectors in stress conditions and many more (Rya et al. 2007; Staniek et al. 2008; Rai et al. 2014a, b). They also possess the potential to produce unique secondary metabolites, which can be exploited in pharmaceutical, agricultural and other industries. Thus, there is a growing interest of researchers in bioprospecting of endophytic microbial communities inhabiting the plants from various ecosystems.

*Actinobacteria* are Gram-positive typically filamentous bacteria, and is a major phylum in the domain* Bacteria* (Ludwig and Klenk 2005). Actinobacteria are widely distributed in both terrestrial and aquatic ecosystems. They play important roles in decomposition of complex materials from dead plants, animals, algae and fungi and in recycling of the nutrients resulting in humus formation (Sharma 2014). Actinobacteria are an important and a large group of soil microbes with high potential of producing different bioactive metabolites including antimicrobial, anticancer and other pharmaceutical compounds (Fiedler et al. 2008; Schulz et al. 2009). These microbes have been the largest producers of different antibiotics since the discovery of Penicillin in 1928 and provided the vast diversity of antibiotics against many deadly diseases. Total number of bioactive metabolites produced by microorganisms are around 23,000 out of which 10,000 (45 % of all bioactive metabolites) are produced by actinobacteria alone and among this group of bacteria, 7600 (76 %) compounds are reported from a single genus *Streptomyces* (Berdy 2012). This signifies their prime importance in the world of pharmaceuticals.

It is well known that the medicinal plants are the rich sources of precious bioactive compounds. As a consequence of long term association of endophytes with such plants, the former may also participate in metabolic pathways and enhance its own natural bioactivity or may gain some genetic information to produce specific biologically active compound similar to the host plant (Stierle et al. 1993; Eyberger et al. 2006; Mitchell et al. 2010; Kumar et al. 2013; Chithra et al. 2014; Rai et al. 2014a, b). Therefore, the endophytes isolated from medicinal plants are of immense significance.

The beneficial interactions of endophytic actinobacteria with plants are being considered as an important area of research. These endophytic actinobacteria are attractive source of novel bioactive compounds and therefore, many research groups are involved in the study of their bioactivities and industrial applications. The present review is focused on the advances in endophytic actinobacteria isolated from medicinal plants including their diversity and broad-spectrum bioactivities.

## Isolation of endophytic actinobacteria

Different methods have been used by researchers for isolation of endophytic actinobacteria. Takahashi and Omura (2003) emphasized that the diversity of actinobacteria depend mainly on the methods of isolation. The most frequently employed method for their detection and enumeration involves isolation from surface-sterilized host plant tissue. Isolation of endophytic actinobacteria depend on various factors, which include- host plant species, age and type of tissue, geographical and habitat distribution, sampling season, surface sterilants, selective media and culture conditions (Hallmann 2001; Gaiero et al. 2013).

In general, the isolation protocol involves the collection of plant parts such as leaves, stem, roots that should be processed freshly or stored at 4 °C until isolation within 24 h. These explants are washed in running tap water to remove adhered epiphytes, soil debris or dust particles on the surface, followed by surface sterilization using one or more different surface sterilizing agents. The most commonly used surface sterilants include ethanol and a strong oxidant or general disinfectant like household bleach (NaOCl) with 2–5 % (w/v), available chlorine (for 2–4 min). Qin et al. (2008b) and Dochhil et al. (2013) applied combination of 5 % sodium chlorate (NaClO_3_), 2.5 % sodium thiosulfate (Na_2_S_2_O_3_), 75 % ethanol and 10 % sodium bicarbonate (NaHCO_3_) as sterilizing agents to inhibit the growth of fungal endophytes. The strength of sterilizing chemicals depends on permeability of the sample. Otherwise, the internal tissues will be sterilized (Hallmann et al. 2006). All the explants are finally rinsed with sterile distilled water, divided into small fragments (1 cm for steam or roots and 1 cm ^2^ for leaves) and inoculated on appropriate agar medium. In another method, the surface sterilized plant tissues are macerated and thoroughly homogenized with phosphate buffer or other suitable liquid medium. This suspension is serially diluted as 10^−1^ to 10^−5^ and spread on agar medium in order to obtain endophytic actinobacteria. The media are supplemented with antifungal antibiotics such as nystatin and cycloheximide (50 or 100 μg/ml) to suppress the fungal growth. After incubation at 26 ± 2 °C for 15–30 days, individual colonies with characteristic actinomycete morphology emerging out from the plant tissue are isolated. The pure cultures of the isolates are obtained by streaking on fresh media plates. The efficacy of the surface sterilization method, resulting from lack of microbial growth, can be authenticated by inoculating the last washing water into the same media plates.

Various types of growth media have been described by the authors for the isolation of endophytic actinobacteria such as starch casein (Küster and Williams 1964), starch casein nitrate (SCNA), actinomycetes isolation, soybean (Williams and Davies 1965), chitin-vitamin B (Hayakawa and Nonomura 1987), tap water-yeast extract (TWYE; Crawford et al. 1993) agars and humic acid vitamin B (HV), yeast extract casamino acid (YECA), synthetic (Mincer et al. 2002), modified Gausse (Ivantiskaya et al. 1978) and glycine–glycerol (Küster 1959) media. Zhao et al. (2011) also underlined the need of using wider range of isolation methods to acquire more knowledge about species diversity of actinobacteria within medicinal plants. A modified method employed by Machavariani et al. (2014) describes the pre-treatment of leaves with solutions of heteroauxin and zircon, which helped to isolate and increase the numbers of rare actinobacteria from medicinal plants.

## Diversity of endophytic actinobacteria in medicinal plants

Current identification and classification of actinobacteria are based a polyphasic approach, comprising morphological, physiological and molecular studies (Goodfellow et al. 2012) based on each taxon should be described and differentiated from related taxa. The sequencing of highly conserved macromolecules, notably 16S rRNA genes, has provided valuable data for constructing phylogenies at and above the genus level (Ludwig and Klenk 2005). The DNA: DNA relatedness, molecular fingerprinting and phenotypic techniques are methods of choice for delineating taxa at and below the rank of species (Rosselló-Mora and Amann 2001). Distinguishing phenotypic differences are required for the description of a new species (Wayne et al. 1987). Exploring the diversity of endophytic actinobacteria is indispensable for screening of beneficial strains and understanding their ecological niche.

Endophytic actinobacteria are able to associate with their host at a very early stage of the plant development (Hasegawa et al. 2006). Minamiyama et al. (2003) noticed in SEM studies that mycelia of *Streptomyces galbus*, which was spread on the surface of the tissue-culture medium in which rhododendron seedlings were growing, grew on leaf surfaces and entered into the leaf tissues via stomata. Further, they also observed that the internal mycelia grew out of stomata after internal multiplication within host leaves. Moreover, the authors observed that within host leaves, hyphae of *S. galbus* were present individually or in colonies in intercellular spaces but not inside epidermal or mesophyll cells.

The maximum endophytic actinobacteria have been recovered from roots followed by stems and least in leaves (Qin et al. 2009; Gangwar et al. 2014). The woody plants conferred far greater diversity of actinobacteria in comparison to herbaceous plants. The high rate of occurrence of actinobacteria in roots as compared to other tissues is very common. This underlines the fact that the actinobacteria are natural dwellers of soil that easily come in contact with the roots of plants and may form the symbiotic association with them by entering the plant tissues. The results obtained by Nimnoi et al. (2010) suggested that different locations within the plant also differ in the diversity of actinomycete flora. Strobel and Daisy (2003) reported that the greater diversity of endophytes is probable to occur in the tropical and temperate regions. Du et al. (2013a) analyzed the endophytic diversity of 37 medicinal plants and reported 600 actinobacteria belonging to 34 genera and 7 unknown taxa. The authors depicted that there was no direct relationship between host plants and their endophytic flora regarding the utilization of sole carbon source, fermentation of carbon sources for production of acids and enzymes, rather the physiological characteristics of endophytic isolates were related to the geographical distribution of their host plants.

The measures of functional biodiversity may be more reliable and powerful than the taxonomic measures in order to recognize mechanistic basis of diversity and its effects on the plant-endophyte interactions (Parrent et al. 2010). Species distribution and biological diversity of endophytic actinobacteria of medicinal plants are extensively influenced by ecological environment (Hou et al. 2009). El-Shatoury et al. (2013) interpreted that the plant species can be separated into three clusters representing high, moderate and low endophytic diversity on the basis of generic diversity analysis of endophytes. The authors also reported that the endophytes represent high functional diversity, based on forty four different traits including catabolic and plant growth promotion traits and such traits may characterize a key criteria for successful habitation of endophytes within the endosphere. Furthermore, the stress-tolerance traits were more predictive measure of functional diversity of endophytic actinobacteria (El-Shatoury et al. 2013).

Hasegawa et al. (1978) reported a new genus of actinobacteria namely *Actinosynnema*, from a grass blade, which was probably the first report of an actinomycete of plant origin. A comprehensive literature survey has revealed the huge diversity of endophytic actinobacteria isolated from interior tissues of stem, leaves and roots of medicinal plants (Table 1). Taechowisan et al. (2003) studied the diversity of actinobacteria residing in medicinal plants based on their morphology and the amino acid composition of the whole-cell extract and analysed the percentage of endophytic actinobacteria recovered from different explants: 64 % isolates from roots, 29 % from leaves, and 6 % from stems of 36 different plant species.

**Table 1:** Endophytic actinobacteria isolated from medicinal plants

Species of actinomycetes	Host plant	Tissue	Bioactive compounds	Reference
*Streptomyces* *longisporoflavus*, *Streptomyces* sp.	*Rauwolfia densiflora*	Stem, leaf, inflorescence	ND	Akshatha et al. (2014)
*Amycolatopsis* sp., *Micromonospora* sp., *Streptomyces* sp.	*Siparuna crassifolia,* *Calycophyllum acreanum,* *Capirona decorticans,* *Ocotea longifolia,* *Aspidosperma* sp, *Palicourea longifolia,* *Monstera spruceana,* *Croton lechleri,* *Cantua buxifolia,* *Banisteriopsis caapi,* *Iryanthera laevis,* *Eucharis cyaneosperma*	Stem	ND	Bascom-Slack et al. (2009)
*Kineococcus endophytica*	*Limonium sinensis*	ND	ND	Bian et al. (2012b)
*Streptomyces phytohabitans*	*Curcuma phaeocaulis*	Root	ND	Bian et al. (2012a)
*Kitasatospora* sp.	*Taxus baccata*	Wood/inner cortical tissues	Paclitaxel	Caruso et al. (2000)
*Streptomyces* sp. NRRL 30562	*Kennedia nigriscans*	Stem	Munumbicins A, B, C and D	Castillo et al. (2002)
*Streptomyces* sp. NRRL 30566	*Grevillea pteridifolia*	Stem	Kakadumycins	Castillo et al. (2003)
*Streptomyces* sp. NRRL 30562	*Kennedia nigriscans*	ND	Munumbicins E-4 and E-5	Castillo et al. (2006)
*Pseudonocardia endophytica*	*Lobelia clavatum*	Inner tissue	ND	Chen et al. (2009)
*Micromonospora* sp., *Nonomuraea* sp., *Planotetraspor* sp., *Pseudonocardia* sp.	*Elaeagnus angustifolia*	Root nodules	ND	Chen et al. (2011)
*Microbispora* sp.	*Spermacoce verticillata*	Leaf	ND	Conti et al. (2012)
*Streptomyces* sp.	*Centella asiatica*	Root, stem, leaf	Indole acetic acid (IAA)	Dochhil et al. (2013)
*Allonocardiopsis opalescens*	*Lonicera maackii*	Fruit	ND	Du et al. (2013b)
*Streptomyces* sp. Hedaya48	*Aplysina fistularis*	Inner healthy tissue	Vanillin, 5,7-dimethoxy-4-*p*-methoxylphenylcoumarin, Saadamycin	El-Gendy and EL-Bondkly (2010)
*Streptomyces* sp.	*Artemisia herba*-*alba*, *Echinops spinosus,* *Mentha longifolia,* *Ballota undulate*	Green aerial parts	ND	El-Shatoury et al. (2006)
*Kibdelosporangium* sp., *Kitasatosporia* sp., *Nocardia* sp., *Nocardioides* sp., *Promicromonospora* sp. *Pseudonocardia* sp., *Streptomyces* sp.	*Achillea fragrantissima*	ND	Siderophores, Chitinase	El-Shatoury et al. (2009)
*Streptomyces* sp. MSU-2110	*Monstera* sp.	Stem	Coronamycin	Ezra et al. (2004)
*Actinopolyspora* sp., *Micromonospora* sp., *Saccharopolyspora* sp., *Streptomyces* sp.	*Aloe vera,* *Mentha,* *Ocimum sanctum*	Root, stem, leaf	ND	Gangwar et al. (2011)
*Actinopolyspora* sp., *Micromonospora* sp., *Saccharopolyspora* sp., *Streptomyces* sp.	*Aloe vera,* *Mentha arvensis,* *Ocimum sanctum*	Root, stem, leaf	Hydroxamate-type of siderophore, Catechol-type of siderophore, Indole acetic acid (IAA)	Gangwar et al. (2014)
*Streptomyces* sp. TP-A0569,	*Allium fistulosum*	Leaf	Fistupyrone 7′-Demethylnovobiocin, 5″-demethylnovobiocin, Novobiocin, 6-Prenylindole, Anicemycin Pteridic acids A and B	Igarashi (2004)
*Streptomyces hygroscopicus* TP-A0451,	*Pteridium aquilinum*	Stem	Clethramycin	
*Streptomyces hygroscopicus* TP-A0326,	ND	ND	Cedarmycins A and B	
*Streptomyces* sp. TP-A0456	*Cryptomeria japonica*	Twig		
*Streptomyces hygroscopicus* TP-A0451	ND	ND	Pterocidin	Igarashi et al. (2006)
*Micromonospora lupini*	ND	ND	Lupinacidins	Igarashi et al. (2007)
*Streptomyces cavourensis* AB184264.1	*Catharanthes roseus*	Leaf	ND	Kafur and Khan (2011)
*Streptomyces laceyi* MS53	*Ricinus communis*	Stem	6-Alkylsalicylic acids (salaceyins A and B)	Kim et al. (2006)
*Actinomycetes* sp.	*Emblica officinalis*	Twig, leaf	ND	Kumar et al. (2011)
*Streptomyces* sp.	*Cistanches deserticola*	Root	Tyrosol (possible ligand for GPR12) Phenylethylamine derivatives, Cyclic dipeptides, Nucleosides and their aglycones, *N*-acetyltryptamine and Pyrrole-2-carboxylic acid	Lin et al. (2008)
*Streptomyces* sp. CS	*Maytenus hookeri*	ND	24-demethyl-bafilomycin C1 (Naphthomycin A)	Lu and Shen (2003)
*Streptomyces* sp. CS	*Maytenus hookeri*	Tissue cultures	Naphthomycin K, A and E	Lu and Shen (2007)
*Micromonospora* sp., *Nocardiopsis* sp., *Streptomyces* sp.	*Achillea millefolium,* *Aloe arborescens,* *Anthoxantum odoratum,* *Arctium lappa,* *Convallaria majalis,* *Fragaria vesca,* *Geranium pretense,* *Hippophae rhamnoides,* *Lysimachia nummularia,* *Matricaria matricarioides,* *Melilotus officinalis,* *Mentha arvensis,* *Plantago major,* *Rosa cinnamomea,* *Rubus idaeus,* *Tanacetum vulgare,* *Taraxacum officinale,* *Trifolium pretense* *Urtica dioica,* *Viola odorata*	Leaf	ND	Machavariani et al. (2014)
*Actinomadura* sp. *Kibdelosporangium* sp., *Kitasatosporia* sp., *Nocardioides* sp., *Pseudonocardia* sp., *Streptomyces* sp., Undefinied actinomycetes	*Phyllanthus niruri,* *Withania somnifera,* *Catharanthus roseus,* *Hemidesmus indicus*	Root	Volatile organic compounds (VOCs), Diffusible metabolites Chitinase, Cellulases, CMC-ase,	Mini Priya (2012)
*Actinomadura* sp., *Kibdelosporangium* sp., *Kitasatosporia* sp., *Nocardia* sp., *Nocardioides* sp., *Pseudonocardia* sp., *Streptomyces* sp., Undefinied actinomycetes	*Achillea fragrantissima,* *Catharanthus roseus,* *Artemisia herba*-*alba,* *A. judaica,* *Jasonia montan,* *Launae* sp., *Echinops spinosissimus,* *Pulicaria* sp., *Centauria* sp., *Nerium oleander*	ND	Volatile organic compounds (VOCs), Diffusible metabolites	Moussa et al. (2011)
*Actinomycete* sp., *Brevibacterium* sp., *Leifsonia* sp., *Microbacterium* sp., *Streptomyce*s sp.	*Mirabilis jalapa,* *Clerodendrum colebrookianu,* *Eupatorium odoratum*, *Alstonia scholaris* *Musa superba*	Leaf, stem, root, flower	ND	Passari et al. (2015)
*Streptomyces olivochromogenes*	*Tinospora crispa* *Phaleria macrocarpa,* *Curcuma aeruginosa,* *Andrographis paniculata,* *Caesalpinia sappan,* *Curcuma xanthoriza,* *Gynura procumbens,* *Physalis peruviana,* *Hibiscus sabdariffa*	Root, leaf, stem	Inhibitor of alpha-glucosidase	Pujiyanto et al. (2012)
*Streptomyces setonii,* *Streptomyces sampsonii,* *Streptomyces* sp. Q21, *Streptomyce*s sp. MaB- QuH- 8	*Maytenu saquifolia*, *Putterlickia retrospinosa*, *Putterlickia verrucosa*	ND	Celastramycins A and B	Pullen et al. (2002)
*Glycomyces endophyticus*	*Carex baccans*	Root	ND	Qin et al. (2008b)
*Glycomyces mayteni* *Glycomyces scopariae*	*Scoparia dulcis,* *Maytenus austroyunnanensis*	Root	ND	Qin et al. (2009)
*Pseudonocardia sichuanensis*	*Jatropha curcas*	Root	ND	Qin et al. (2011)
*Nocardioides panzhihuaensis*	*Jatropha curcas*	Stem	ND	Qin et al. (2012a)
*Actinomadura* sp., *Amycolatopsis* sp, *Cellulosimicrobium* sp., *Glycomyces* sp., *Gordonia* sp., *Janibacter* sp., *Jiangella* sp., *Microbacterium* sp., *Micromonospora* sp., *Mycobacterium* sp., *Nocardia* sp., *Nocardiopsis* sp., *Nonomuraea* sp., *Plantactinospora* sp, *Polymorphospora* sp., *Promicromonospora* sp., *Pseudonocardia* sp., *Saccharopolyspora* sp, *Streptomyces* sp, *Streptosporangium* sp., *Tsukamurella* sp.	*Maytenus austroyunnanensis*	Root, stem, leaf	ND	Qin et al. (2012b)
*Streptomyces* sp.	*Azadiracta indica,* *Ocimum sanctum,* *Phyllanthus amarus*	Root, leaf	ND	Shenpagam et al. (2012)
*Streptomyces antibioticus*	*Curcuma domestica,* *Phaleria macrocarpa,* *Isotoma longiflora,* *Symplocos cochinensis*	Root, stem, leaf	ND	Sunaryanto and Mahsunah (2013)
*Streptomyces aureofaciens* CMUAc130	*Zingiber officinale,* *Alpinia galanga*	Root	ND	Taechowisan and Lumyong, (2003)
*Microbispora* sp, *Micromonospora* sp., *Nocardia* sp., *Streptomyces* sp., Unidentified isolates	*Zingiber officinale*, *Alpinia galanga*	Root, stem, leaf	ND	Taechowisan et al. (2003)
*Streptomyces aureofaciens* CMUAc130	*Zingiber officinale*	Root	5,7- dimethox y-4-*p*-methoxylphenylcoumarin, 5,7-dimethoxy-4-phenylcoumarin	Taechowisan et al. (2005, 2007)
*Microbispora* sp., *Micromonospora* sp. *Nocardia* sp., *Streptomyces* sp. Tc022, Unidentified isolates	*Alpinia galanga*	Root	Actinomycin D	Taechowisan et al. (2006)
*Microbispora* sp., *Nocardia* sp., *Sacchromonospora* sp., *Streptomyces* sp., *Streptosporangium* sp., *Streptoverticillium* sp.	*Azadirachta indica*	Root, stem, leaf,	ND	Verma et al. (2009)
*Jishengella endophytica* 161111	*Xylocarpus granatum*	Root	Alkaloids	Wang et al. (2014)
*Saccharopolyspora dendranthemae*	*Dendranthema indicum*	Stem	ND	Zhang et al. (2013)
*Streptomyces* sp. neau-D50	Soybean	Root	3-acetonylidene-7-prenylindolin-2-one (isoprenoids, 7-isoprenylindole-3-carboxylic acid, 3-cyanomethyl-6-prenylindole, 6-isoprenylindole-3-carboxylic acid and 7,40 -dihydroxy-5-methoxy-8-(g,g-dimethylallyl)-flavanone)	Zhang et al. (2014)
*Micromonospora* sp., *Nonomuraea* sp., *Oerskovia* sp., *Promicromonospora* sp., *Rhodococcus* sp., *Streptomyces* sp.	*Potentilla discolor,* *Ainsliaea henryi*, *Impatiens chinensis*, *Rhizoma Arisaematis*, *Dioscorea opposita*, *Stellera chamaejasme*, *Salvia miltiorrhiza*, *Drosera peltata*, var.multisepala, *Artemisia annua*, *Achyranthes aspera*, *Cynanchum auriculatum*, *Gnaphalium hypoleucum*, *Mosla dianthera*, *Cassytha filiformis*, *Vaccinium bracteatum*	Root, stem, leaf	ND	Zhao et al. (2011)
*Streptomyces* sp. YIM66017	*Alpinia oxyphylla*	ND	2,6-dimethoxy terephthalic acid, yangjinhualine A, α-hydroxyacetovanillone and cyclo(Gly-Trp)	Zhou et al. (2014)

*ND* no data

Janso and Carter (2010) also assessed the diversity of endophytic actinobacteria, including those from medicinal plants, albeit by ribotyping with Pvu II restriction enzyme to digest the genomic DNA. Ribotypes were then compared to each other using appropriate software (Janso and Carter 2010). The authors have found that 85 % of 123 isolates studied were determined to be unique at the strain level. The isolates were classified to six families and 17 different genera. *Streptomyces* accounts for the dominant genus, which is most commonly isolated as endophytic actinomycete (Qin et al. 2009; Zhao et al. 2011; Shutsrirung et al. 2014; Gangwar et al. 2014) while others include genera such as *Micromonospora*, *Actinopolyspora*, *Saccharopolyspora, Nocardia*, *Oerskovia*, *Nonomuraea*, *Streptoverticillium*, *Microbispora*, *Streptosporangium*, *Promicromonospora* and *Rhodococcus* (Verma et al. 2009; Zhao et al. 2011). Some rare actinobacteria like *Dietzia*, *Blastococcus*, *Dactylosporangium*, *Actinocorallia*, *Jiangella,Promicromonospora*, *Oerskovia*, *Microtetraspora* and *Intrasporangium* were also reported as endophytes (Qin et al. 2009; Zhao et al. 2011; Qin et al. 2012b; El-Shatoury et al. 2013). A novel halotolerant actinomycete was isolated from a salt marsh plant *Dendranthemaindicum* collected from the coastal region of China (Zhang et al. 2013). New species of endophytic actinobacteria such as *Rhodococcuscercidiphylli* and *Saccharopolysporaendophytica* were isolated from leaf of *Cercidiphyllum japonicum* (Li et al. 2008) and root of *Maytenus austroyunnanensis* (Qin et al. 2008a), respectively. Du et al. (2013b) proposed a new genus and species, *Allonocardiopsis opalescens* gen. nov., sp. nov., based on the polyphasic taxonomic study, within the suborder *Streptosporangineae*. Wang et al. (2008) studied the diversity of uncultured microbes associated with medicinal plant *Mallotus nudiflorus* and concluded that actinobacteria were the most dominant microbes, covering about 37.7 % of whole endophytic isolates.

In 2012b, Qin and co-workers studied the diversity of endophytic actinobacteria recovered from root, stem and leaf tissues of *Maytenus austroyunnanens* which was collected from tropical rainforest in Xishuangbanna, China. Later the authors concluded the diversity of isolates by combination of cultivation and culture-independent analysis and based on 16S rRNA gene sequencing. Further by using different selective isolation media and methods total of 312 actinobacteria were isolated from above plants which were affiliated with the order *Actinomycetales* (distributed into 21 genera). Based on a protocol for endophytes enrichment, three 16S rRNA gene clone libraries were constructed and 84 distinct operational taxonomic units were identified and they distributed among the orders *Actinomycetales* and *Acidimicrobiales*, including eight suborders and at least 38 genera with a number of rare actinobacteria genera. Moreover, six genera from the order *Actinomycetales* and uncultured clones from *Acidimicrobiales* were found to be unknown and reported as first time endophytes. This study confirms abundant endophytic actinobacterial consortium in tropical rainforest native plant and suggests that this special habitat still represents an underexplored reservoir of diverse and novel actinobacteria of potential interest for bioactive compounds discovery.

## Bioactivities of endophytic actinobacteria

The plant endosphere consists of a large variety of microbial endophytes, which constitute a complex micro-ecosystem (El-Shatoury et al. 2013). A vast diversity of secondary metabolites in actinobacteria may occur due to the natural adaptations to environment, as a part of competition for common resources such as plant matter in soil. It has been observed that the genes responsible for the production of individual secondary metabolites were found almost always located as a cluster in the genome and referred to as biosynthetic gene clusters (Doroghazi and Metcalf 2013). Although, there is no data available about full genome sequencing on actinobacteria from medicinal plants it has been known, that whole genomes of *Streptomyces* sp. and non-*Streptomyces* non-endophytic actinobacteria such as *Streptomyces avermitilis* MA-4680 (Ōmura et al. 2001; Ikeda et al. 2003) and *Streptomyces coelicolor* A(3)2 (Bentley et al. 2002) as well as *Saccharopolyspora erythraea* NRRL 23338 (Oliynyk et al. 2007), *Salinispora tropica* CNB-440 (Udwary et al. 2007) contain around 20 or more natural product biosynthetic gene clusters for the production of known or predicted secondary metabolites (Goodfellow and Fiedler 2010). The potential of actinobacteria isolated from medicinal plants, especially of non-productive non-streptomycete ones to produce secondary metabolites can be estimated by detection of polyketide synthase (PKS) (both I and II type) and nonribosomal peptide synthetase (NRPS) genes (Janso and Carter 2010). The authors studied 29 strains and all of them produced bands of the expected size for NPRS and majority of them possessed PKS (66 % of PKSI and 79 % of PKSII type) genes. However, some of the pathways encoded by these genes may not be functional. The above study suggests that the non-productive actinobacteria possess the genetic capacity to produce secondary metabolites, if cultivated under proper growth conditions (Janso and Carter 2010).

Amongst prokaryotes, members of Actinobacteria, notably the genus *Streptomyces*, remains the richest source of valuable natural products (Pandey et al. 2004; Newman and Cragg 2007; Lu and Shen 2007; Olano et al. 2009; Berdy 2012). The diverse arrays of bioactivities of endophytic actinobacteria are further classified into pharmaceutical and agricultural applications and are illustrated below in detail.

## Pharmaceutical applications

### Antimicrobial and antiviral activity

In recent years, many of novel antibiotics synthesized by endophytic actinobacteria recovered from medicinal plants found to be active against bacteria, fungi and viruses. Moreover, these antibiotics demonstrated their activity at significantly lower concentrations (Table 2). This indicates the strong and broad spectrum microbiocidal potential of the antibiotics originating from endophytic actinobacteria, mainly of the genus *Streptomyces*.

**Table 2:** Bioactivity of compounds from endophytic actinobacteria isolated from medicinal plants

Compound	Target cells/microorganism	MIC (µg ml^−1^)	Reference
Munumbicins A, B, C and D from *Streptomyces* sp. NRRL 30562	*Pseudomonas aeruginosa*	–	Castillo et al. (2002)
	*Vibrio fischeri*	–	
	*Enterococcus faecalis*	–	
	*Staphylococcus aureus*	–	
	*Acinetobacter* sp.	–	
	*Neisseria gonorrhoeae*	–	
	*Streptococcus pneumoniae*	–	
	*Bacillus anthracis*	–	
	*Escherichia coli*	–	
	*Pythium ultimum*	0.2–4.0	
	*Rhizoctonia solani*	1.5–15.6	
	*Phytophthora cinnamomi*	1.5–15.6	
	*Geotrichum candidum*	15.5–31.2	
	*Sclerotinia sclerotiorum*	0.2–8.0	
	*Pseudomonas syringe*	0.2–15.6	
	*Cryptococcus neoformans*	10	
	*Candida albicans*	10	
	*Aspergillus fumigates*	20	
	*Staphylococcus aureus* ATCC 33591 (methicillin resistant)	No activity–2.5	
	*Staphyloccus aureus* MH II (vancomycin sensitive)	0.4	
	*Enterococcus faecalis* ATCC 51299	No activity–16	
	*Mycobacterium tuberculosis* MDR-P (drug resistant)	10–125	
	*Mycobacterium tuberculosis* H37Rv (ATCC 25618) (drug sensitive)	46–150	
Kakadumycin A from *Streptomyces* sp. NRRL 30566	*Bacillus anthracis* 40/BA 100	0.3	Castillo et al. (2003)
	*Bacillus anthracis* 14578	0.55	
	*Bacillus anthracis* 28	0.43	
	*Bacillus anthracis* 62-8	0.41	
	*Staphylococcus simulans* ATCC 11631	0.25	
	*Enterococcus faecalis* ATCC 29212	0.062	
	*Enterococcus faecalis* VRE, ATCC 51299	0.062	
	*Enterococcus faecium* ATCC 49624	0.062	
	*Listeria monocytogenes* ATCC 19114	0.25	
	*Listeria monocytogenes* ATCC 19115	0.25	
	*Shigella dysenteriae* ATCC 11835	4.0	
	*Staphylococcus epidermidis* ATCC 12228	0.125	
	*Staphylococcus aureus* ATCC 29213	0.125	
	*Staphylococcus aureus* MRSA, ATCC 33591	0.5	
	*Staphylococcus aureus* GISA, ATCC 700787	0.5	
	*Staphylococcus aureus* ATCC 27734	0.125	
	*Streptococcus pneumoniae* ATCC 49619	<0.0325	
	*Streptococcus pneumoniae* ATCC 70674	<0.0325	
	*Streptococcus pneumoniae* ATCC 70676	<0.0325	
	Inhibitor of human breast cancer cell line BT20	n/a	
Munumbicins E-4 and E-5 from *Streptomyces* sp. NRRL 30562	*Burkholderia thailandensis*	192–256	Castillo et al. (2006)
	*Escherichia coli*	16	
	*Staphylococcus aureus* ATCC 29213	4–8	
	*Staphylococcus aureus* 43000 (MRSA)	8–16	
	*Staphylococcus aureus*	32	
	*Pythium ultimum*	5	
	*Bacillus subtilis*	5	
	*Rhizoctonia solani*	80	
	Cytotoxic activity against *Plasmodium falciparum*	n/a	
Saadamycin/5,7-Dimethoxy-4-p-methoxylphenyl coumarin from *Streptomyces* sp. Hedaya48	*Trichophyton rubrum*	5,0/7.5	El-Gendy and EL-Bondkly (2010)
	*Trichophyton mentagrophytes*	1.5/90	
	*Microsporum gypseum*	1.25/100	
	*Epidermophyton floccosum*	1.0/50	
	*Aspergillus niger*	1.0/20	
	*Aspergillus fumigates*	1.6/10	
	*Fusarium oxysporum*	1.2/22	
	*Candida albicans,*	2.22/15	
	*Cryptococcus humicolus*	5.15/10	
Coronamycin from *Streptomyces* sp. MSU-2110	*Pythium ultimum*	2	Ezra et al. (2004)
	*Phytophthora cinnamomi*	16	
	*Aphanomyces cochlioides*	4	
	*Geotrichum candidum*	>500	
	*Aspergillus fumigates*	>500	
	*Aspergillus ochraceus*	>500	
	*Fusarium solani*	>500	
	*Rhizoctonia solani*	>500	
	*Cryptococcus neoformans* (ATCC 32045)	4	
	*Candida parapsilosis* (ATCC 90018)	>32	
	*Candida albicans* (ATCC 90028)	16–32	
	*Saccharomyces cerevisiae* (ATCC 9763)	>32	
	*Candida parapsilosis* (ATCC 22019)	>32	
	*Candida albicans* (ATCC 24433)	>32	
	*Candida krusei* (ATCC 6258)	>32	
	*Candida tropicalis* (ATCC 750)	>32	
6-prenylindole from *Streptomyces* sp. TP-A0595	*Alternaria brassicola*	Data not given	Igarashi (2004)
Fistupyrone from *Streptomyces* sp. TP-A0569	Suppressing spore germination of *Alternaria brassicicola*	n/a	
Clethramycin from *Streptomyces hygroscopicus* TP-A0326	*Candida albicans* *Cryptococcus neoformans*	1.0 1.0	
Cedarmycin from *Streptomyces* sp. TP-A0456	*Candida glabrata*	0.4	
Anicemycin from *Streptomyces thermoviolaceus* TP-A0648	Cytocidal activity against tumor cell lines	n/a	
Pterocidin from *Streptomyces hygroscopicus* TP-A0451	Cytotoxicity against human cancer cell lines NCI-H522, OVCAR-3, SF539, and LOX-IMVI	n/a	Igarashi et al. (2006)
Lupinacidins A and B from *Micromonospora lupini* sp.	Inhibitor of in vitro invasion of colon 26-L5 cells	n/a	Igarashi et al. (2007)
6-Alkalysalicyclic acids (Salaceyins A and B) from *Streptomyces laceyi* MS53	Cytotoxicity against human breast cancer cell line SKBR3	n/a	Kim et al. (2006)
Naphthomycin K from *Streptomyces* sp. CS	*Penicillium avellaneum* UC-4376	–	Lu and Shen (2003, 2007)
	*Staphylococcus aureus*		
	*Mycobacterium tuberculosis*		
	Cytotoxicity against P388 and A-549 human tumor cells	n/a	
Celastramycins A/B from *Streptomyce*s MaB- QuH- 8	*Staphylococcus aureus* MRSA 134/93	0.1/no activity	Pullen et al. (2002)
	*Staphylococcus aureus* MR 994/93	0.2/no activity	
	*Enterococcus faecalis* V-r 1528	0.8/no activity	
	*Mycobacterium smegmatis* SG 987	1.6/no activity	
	*Mycobacterium aurum* SB 66	0.4/no activity	
	*Mycobacterium vaccae IMET 10670*	0.05/no activity	
	*Mycobacterium fortuitum*	3.1/no activity	
	*Bacillus subtilis* ATCC 6633	0.05/no activity	
5,7-dimethoxy-4-pmethoxylphenylcoumarin; 5,7-dimethoxy-4-phenylcoumarin from *Streptomyces aureofaciens* CMUAc130	*Colletorichum musae*	120 150	Taechowisan et al. (2005)
Actinomycin D from *Streptomyces* sp. Tc022	*Colletotrichum musae* *Candida albicans*	10 20	Taechowisan et al. (2006)
5,7-Dimethoxy-4-pmethoxylphenylcoumarin; 5,7-dimethoxy-4-phenylcoumarin from *Streptomyces aureofaciens* CMUAc130	Antitumor activity	n/a	Taechowisan et al. (2007)
Perlolyrine, 1-hydroxy-β-carboline, lumichrome, 1*H*-indole-3-carboxaldehyde from *Jishengella endophytica* 161111	Antiviral activity	n/a	Wang et al. (2014)
3-Acetonylidene-7-prenylindolin-2-one (isoprenoids, 7-isoprenylindole-3-carboxylic acid, 3-cyanomethyl-6-prenylindole, 6-isoprenylindole-3-carboxylic acid and 7,40 -dihydroxy-5-methoxy-8-(g,g-dimethylallyl)-flavanone) from *Streptomyces* sp. neau-D50	Cytotoxic activity against human lung adenocarcinoma cell line A549 *Colletotrichum orbiculare,* *Phytophthora* *capsici,* *Corynespora cassiicola,* *Fusarium oxysporum*	n/a	Zhang et al. (2014)
2,6-Dimethoxy terephthalic acid, yangjinhualine A, a-hydroxyacetovanillone, cyclo(Gly-Trp) from *Streptomyces* sp. YIM66017	Antioxidant activity	n/a	Zhou et al. (2014)

(–) not tested, *n/a* not applicable

Day by day due to excessive use of antibiotics, the multi-drug resistance capacity of pathogens is becoming more and more severe. The scientists all over the world are endeavouring continuously to search new antibiotic compounds in order to tackle this problem. Here endophytic microbes, especially actinobacteria appear as a source of novel and active compounds to combat the increasing number of multidrug-resistant pathogens. Out of 65 strains of endophytic actinobacteria 12 strains were able to suppress penicillin-resistant *Staphylococcus aureus,* belonging to the genus *Glycomyces* and majority of them were *Streptomyces* isolated from plants *Achyranthes bidentata*, *Paeonia lactiflora*, *Radix platycodi* and *Artemisia argyi* (Zhang et al. 2012). Wang et al. (2014) displayed moderate antiviral activity against influenza virus type A subtype H1N1 of perlolyrine, 1-hydroxy-β-carboline, lumichrome, 1*H*-indole-3-carboxaldehyde from *Jishengella endophytica* with IC_50_ value of 38.3, 25.0, 39.7, and 45.9 μg ml^−1^, respectively. Further, they also suggested that 1-hydroxy-β-carboline could be a promising new hit for anti-H1N1 drugs.

### Larvicidal and antimalarial activity

Larvicidal activity of *Streptomyces* sp. isolated from *Artemisia herba*-*alba, Echinops spinosus, Balotta undulate* and *Mentha longifolia* was observed by El-Shatoury et al. (2006). The authors studied cytotoxic effect against larvae of *Artemia salina* was positive for 27 out of 41 endophytic actinobacteria and of these, nine isolates, mainly from *Artemisia* and *Echinops* exhibited high mortality rate reaching to 100 % death after 12 h. Similarly, *Streptomyces albovinaceus* and *S. badius* isolated from plants of family *Asteraceae* were also found to have significant larvicidal potential against first and fourth instar stages of *Culexquinquefasciatus* (mosquito larvae) (Tanvir et al. 2014). They illustrated strong larvicidal activity (80–100 % mortality) of six isolates while four isolates showed potent larvicidal activity (100 % mortality) at the fourth instar stage.

Castillo et al. (2002) have found that one of the tested munumbicins type D was considerably active against the parasite *Plasmodium falciparum*, the most pathogenic plasmodium causing malaria, with IC_50_ of 4.5 ng ml^−1^. They also described that outstanding activity of each of the munumbicins against *P*. *falciparum* were within the range to be pharmacologically interesting with IC_50_ of 175,130, 6.5 and 4.5 ng ml^−1^in munumbicin A–D, respectively. Authors emphasized special interest of the munumbicins C and D because of their extremely low IC_50_ values. Furthermore, they also reported that munumbicins B, C and D did not cause any detectable lysis of human red blood cells up to a concentration of 80 µg ml^−1^. Therefore, they suggested that the ultimate development of these compounds as antimalarial or anti-infectious drugs may have to depend upon the synthesis of munumbicin derivatives that have reduced toxicity (Castillo et al. 2002, 2006).

### Cytotoxicity

Among the range of bioactive compounds from endophytic actinobacteria of medicinal plants those with anticancer activity were also found. Castillo et al. (2003) extracted kakadumicin A, which inhibited the human breast cancer cell line BT20 with IC_50_ of 4.5 ng ml^−1^. Similarly, Igarashi et al. (2006) reported that human cancer cell lines NCI-H522, OVCAR-3, SF539, and LOX-IMVI were inhibited with IC_50_ in the presence of 2.9, 3.9, 5.0 and 7.1 mM of pterocidin extracted from *Streptomyces hygroscopicus* TP-A0451 isolated from *Pteridium aquilinum.* Lu and Shen (2003; 2007) reported cytotoxic activity of naphtomycin A from *Streptomyces* sp. CS isolated from *Maytenus hookeri* against P388 and A549 human tumor cells with IC_50_ 0.07 and 3.17 mM, respectively. The cytotoxicity against A549 human tumor cells was also studied by Zhang et al. (2014). The cell line was inhibited with value of 3.3 and 5.1 mg ml^−1^ in presence of 3-acetonylidene-7-prenylindolin-2-one and 7-isoprenylindole-3-carboxylic acid, respectively. Cytotoxic activity of 6-alkalysalicilic acids, salaceyins A and B from *Streptomyces laceyi* MS53 against human breast cancer cell line, SKBR3 with IC_50_ values of 3.0 and 5.5 mg ml^−1^ was noticed by Kim et al. (2006). Anthraquinones named lupinacidins from *Micromonospora lupine* sp. were reported to inhibit growth of colon 26-L5 carcinoma cells in mice (Igarashi et al. 2007). Furthermore, anti-invasive effects of lupinacidins were also examined at non-cytotoxic concentrations. The authors reported lupinacidin A as more potent both in cytotoxic and anti-invasive activities than lupinacidin B, suggesting that the alkyl substituent present in lupinacidin A was involved in these activities (Igarashi et al. 2007).

Caruso et al. (2000) reported an anticancerous drug paclitaxel from endophytic actinomycete *Kitasatospora* sp. isolated from inner cortical tissues of *Taxus baccata*. Another novel anticancer compound named brartemicin, a trehalose-derived metabolite, was extracted from the actinomycete *Nonomuraea* sp. isolated from *Artemisiavulgaris*. This new compound was capable of inhibiting the invasion of murine colon carcinoma 26-L5 cells with an IC_50_ value of 0.39 μM without any cytotoxicity (Igarashi et al. 2009). Taechowisan et al. (2007) evaluated 4-phenylcoumarins on human lung cancer cell lines, which was extracted from *Streptomyces aureofaciens* and found that 5,7-dimethoxy-4-phenylcoumarin can inhibit cell proliferations more actively when compared with 5,7-dimethoxy-4-p-methoxylphenylcoumarin. Moreover, the screening of 4-arylocoumarins for inhibitory effect on transplanted Lewis lung carcinoma (LLC) by intraperitoned administration has showed antitumor activity with T/C values of 80.08 and 50.0 % at doses of 1 and 10 mg kg^−1^ of 5,7-dimethoxy-4-p-methoxylphenylcoumarin and 81.5 and 44.9 % at doses of 1 and 10 mg kg^−1^ of 5,7-dimethoxy-4-phenylcoumarin. Authors have concluded that 5,7-dimethoxy-4-phenylcoumarin might be preventing or delaying formation of metastases and both 4-arylocoumarins by their low cytotoxicity to normal cells and effect in malignant cells could be recommended as chemopreventatives and in combined antitumor treatment (Taechowisan et al. 2007).

### Antidiabetics

Another important group of compounds, which were found in endophytic actinobacteria from medicinal plants were alpha-glucosidase inhibitors (Pujiyanto et al. 2012). Twelve out of 65 isolates obtained from *Tinospora crispa*, *Caesalpinia sappans* and *Curcuma aeruginosa* were able to produce it. This inhibitor showed antidiabetic property by which it can retard the release of glucose from dietary complex carbohydrates and also delay absorption of glucose. Interestingly, it was observed that endophytic actinomycete BWA65 produced these inhibitors which showed doubled activity than its host plant (*Tinospora crispa*). Furthermore, the tissue cultured plants that were devoid of any endophyte had very low capability to produce inhibitor compounds (Pujiyanto et al. 2012). This indicates that the production of alpha-glucosidase inhibitors by this plant is largely due to the contribution of its endophytic actinobacteria. It also strengthens the hypothesis that there may be a phenomenon of inter-kingdom genetic transfer of some specific traits between the host plant and its endophytic counterpart.

Similarly, Akshatha et al. (2014) isolated alpha-amylase inhibitor secreting endophytic actinobacteria *S. longisporoflavus* and *Streptomyces* sp. from well-known antidiabetic medicinal plants *Leucas ciliata* and *Rauwolfia densiflora*. Alpha-amylase inhibitors demonstrated antidiabetic activity similar to alpha-glucosidase inhibitors. The extracts obtained from these actinobacteria did not show insulin-releasing ability, instead it improved the ability of available insulin to pass glucose into muscles.

### Other bioactive compounds

Phenolic compounds are known as natural antioxidants, which provide protection by scavenging harmful free radicals. Endophytic *Streptomyces* sp. isolated from *Alpiniaoxyphylla* produced two active compounds 2,6-dimethoxy terephthalic acid and yangjinhualine A, which demonstrated considerable antioxidant activity (Zhou et al. 2013; 2014). Out of the total endophytic actinobacteria isolated from medicinal plants, 66.6 % isolates demonstrated potent antioxidant activity (Tanvir et al. 2014). Antiinflammatory drugs are used to reduce the inflammations and this property was also shown by one of the endophytic actinomycete. Taechowisan et al. (2006) demonstrated the successful application of 5,7-dimethyloxy-4-*p*-methoxylphenylcoumarin and 5,7-dimethoxy-4-phenylcoumarin produced by *Streptomyces aureofaciens* as an antiinflammatory agents.

## Agricultural applications

### Plant growth promoters

The endophytic actinomyctetes can also be a source of metabolites, which promote or improve host plant growth as well as reduce disease symptoms caused by plant pathogens or various environmental stresses (Shimizu 2011). Several scientific investigations evidenced the plant growth promotion activity and secretion of plant growth hormones from endophytic actinobacteria. Dochhil et al. (2013) demonstrated the plant growth enhancement and higher seed germination percentage by the application of two *Streptomyces* sp. isolated from *Centella asiatica*. These strains were also evaluated for production of a plant growth promoter, indole acetic acid (IAA) which was found in much higher concentration as 71 g/ml and 197 g/ml. The isolates of the genus *Nocardiopsis* presented highest IAA production ability among all other actinomycete genera (Shutsrirung et al. 2014). In the field trials conducted by El-Tarabily et al. (2010), *Actinoplanes campanulatus*, *Micromonospora chalcea* and *Streptomyces spiralis* were applied individually and in combination to cucumber seedlings, which enhanced plant growth and yield.

Igarashi (2004) and Igarashi et al. (2002) isolated pteridic acids A and B from *Streptomyces hygroscopicus* isolated from a stem of bracken (*Pteridium aquilinum*) as plant growth promoters with auxin-like activity. They found that pteridic acids induced the formation of adventitious roots in hypocotyl of kidney beans at 1 mM as effectively as auxin (indole acetic acid; IAA), a natural plant growth hormone. Additionally, authors noticed that pteridic acid A promotes the root elongation at 20 ppm. However, the rice germination was inhibited at 100 ppm of IAA. Gangwar et al. (2014) also found actinobacteria, mostly *Streptomyces* sp, capable of producing IAA. Plant growth promoters were produced within the range of 9.0–38.8 μg ml^−1^.

Endophytic actinobacteria are able to employ additional means of fungal antagonism such as chitin enzymes and siderophores. Chitin is the most characteristic polysaccharide of the fungal cell wall. Endophytic actinobacteria are able to produce fungal cell wall degrading enzymes especially by the production of chitinase (El-Tarabily and Sivasithamparam 2006). The role of siderophores produced by endophytic microorganisms has been paid more attention because these metabolites are suggested to be involved in promoting the growth of host plants as well as antagonism to phytopathogen (Cao et al. 2005; Tan et al. 2006; Rungin et al. 2012). El-Shatoury et al. (2009) reported actinobacteria from *Achilleafragrantissima*which were either capable of producing chitinases or siderophores and also showed remarkable inhibitory activity against phytopathogenic fungi. Chitinases produced by the endophytic actinomycete *Actinoplanes missouriensis* (El-Tarabily 2003; El-Tarabily and Sivasithamparam 2006) were reported to cause hyphal lysis and reduction in conidial germination. The studies by El-Shatoury et al. (2009) were supported by Gangwar et al. (2014) where authors recorded production of hydroxamate-type of siderophore ranging between 5.9 and 64.9 μg ml^−1^ and catechol-type of siderophore in the range of 11.2–23.1 μg ml^−1^ by actinobacteria from *Aloe vera, Mentha arvensis* and *Ocimum sanctum*. In another investigation, El-Tarabily et al. (2010) applied endophytic *Actinoplanes campanulatus*, *Micromonospora chalcea* and *Streptomyces spiralis* to cucumber seedlings. As it reduced seedling damping-off as well as root- and crown- rot of mature cucumber plants caused by *Pythium aphanidermatum* successfully, authors suggested that these strains of endophytic actinobacteria can be employed as biological control agents.

The 6-prenylindole, a new bioactive compound from *Streptomyces* sp. was studied by Igarashi (2004). This simple molecule showed significant antifungal activity against plant pathogens, *Alternariabrassicicola* and *Fusarium oxysporum*. 6-prenylindole was first reported as a component of the liverwort (*Hepaticae*). This is an interesting example of the isolation of the same compound from plant and microorganism (Igarashi 2004). Similarly, Zhang et al. (2014) showed antifungal activity of one new prenylated indole derivative and tree known hybrid isoprenoids with IC_50_ values in range of 30.55–89.62 against phytopathogenic fungi *Colletotrichumorbiculare, Phytophthora capsici, Corynespora cassiicola* and *Fusarium oxysporum.* Lu and Shen (2003; 2007) reported antifungal activity of naphthomycins A and K extracted from *Streptomyces* sp. CS against *Penicillium avellaneum* UC-4376. Igarashi (2004) reported the compound fistupyrone from *Streptomyces* sp. isolated from a leaf of spring onion (*Allium fistulosum*) and determined as an inhibitor of spore germination of *Alternaria brassicicola.* The latter is the cause of black leaf- spot, a major disease of cultivated *Brassica* plant. Although fistupyrone did not show in vitro antifungal activity against *A. brassicicola*, it completely inhibited the infection of *A. brassicicola* by pretreating the seedlings with 100 ppm of the compound. Studies by Igarashi et al. (2002) revealed that fistupyrone did not give any effect on the growing hyphae but specifically suppresses the spore germination at 0.1 ppm.

Thus, the metabolites obtained from these actinobacteria inhibit the phytopathogenic fungi and can be better and safer alternatives to the chemical fungicides, which pose potential environmental threat and mammalian toxicities. In terms of the availability, the endophytic actinobacteria are the rich and cost-effective source of numerous agro-based biological agents. So, it is desirable to evaluate more such compounds that might have different modes of action to protect the crops than the existing chemical fungicides and will also avoid the problems of cross-resistance.

## Conclusion and future perspectives

There is a pressing need to search for new therapeutic drugs, particularly anti-infective compounds due to the rapid increase of resistance in major known pathogens to front line antibiotics. Therefore, screening and isolation of promising strains of endophytic actinobacteria with antimicrobial properties which are relatively poorly investigated has increased the interest of researchers in both basic and applied fields. Clearly, more research on the formulation, development of novel technologies and methodologies is needed for employing them in the agricultural, medical and pharmaceutical fields.

An extensive characterization and identification of the diverse population of endophytic actinobacteria associated with medicinal plants may also provide greater insight into the plant-endophyte interaction and evolution of mutualism. It is also important to understand the mechanism that enables these microbes to interact with their host plants may be of biotechnological potential. Several questions are yet to be answered. Is there any combination between the metabolic pathways of plants and endophytes, which together constitutes for particular bioactivity? What genetic control exists for synthesis of secondary metabolites similar to the host plants? In order to address this research area in depth, it is necessary to understand the physiology and biochemistry of endophytic actinobacteria as well as their defensive role and secondary metabolite producing ability inside the plants.

## References

[CR1] Akshatha VJ, Nalini MS, D’Souza C, Prakash HS (2014). *Streptomycete* endophytes from anti-diabetic medicinal plants of the Western Ghats inhibit alpha-amylase and promote glucose uptake. Lett Appl Microbiol.

[CR108] Bascom-Slack CA, Ma C, Moore E, Babbs B, Fenn K, Greene JS, Hann BD, Keehner J, Kelley-Swift EG, Kembaiyan V, Lee SJ, Li P, Light DY, Lin EH, Schorn MA, Vekhter D, Boulanger LA, Hess WM, Vargas PN, Strobel GA, Strobel SA (2009) Multiple, novel biologically active endophytic actinomycetes isolated from upper Amazonian rainforests. Microbiol Ecol 58:374–383 10.1007/s00248-009-9494-z 19252940

[CR2] Bentley SD, Chater KF, Cerdano-Tarraga AM, Challis GL, Thomson NR, James KD (2002). Complete genome sequence of the model actinomycete *Streptomyces coelicolor* A3(2). Nature.

[CR3] Berdy J (2012). Thoughts and facts about antibiotics: where we are now and where we are heading. J Antibiot.

[CR109] Bian G-K, Qin S, Yuan B, Zhang Y-J, Xing K, Ju X-J, Li W-J, Jiang J-H (2012a) *Streptomyces phytohabitans* sp. nov., a novel endophytic actinomycete isolated from medicinal plant *Curcuma phaeocaulis*. Ant van Leeuw 102:289–296 10.1007/s10482-012-9737-8 22527624

[CR110] Bian G-K, Feng ZZ, Qin S, Xing K, Wang Z, Cao CL, Liu CH, Dai CC, Jiang J-H (2012b) *Kineococcus endophytica* sp. nov., a novel endophytic actinomycete isolated from a coastal halophyte in Jiangsu. Ant van Leeuw 102(4):621–628 10.1007/s10482-012-9757-4 22669199

[CR4] Cao L, Qiu Z, You J, Tan H, Zhou S (2005). Isolation and characterization of endophytic *Streptomyces* antagonists of *Fusarium* wilt pathogen from surface sterilized banana roots. FEMS Microbiol Lett.

[CR5] Caruso M, Colombo AL, Fedeli L, Pavesi A, Quaroni S, Saracchi M (2000). Isolation of endophytic fungi and actinomycetes taxane producers. Ann Microbiol.

[CR6] Castillo U, Strobel G, Ford E, Hess W, Porter H, Jensen J (2002). Munumbicins, wide- spectrum antibiotics produced by *Streptomyces* NRRL 30562, endophytic on *Kennedia nigriscans*. Microbiol.

[CR7] Castillo U, Harper J, Strobel G, Sears J, Alesi K, Ford E (2003). Kakadumycins, novel anti biotics from *Streptomyces* sp. NRRL 30566, an endophyte of *Grevillea pteridifolia*. FEMS Microbiol Lett.

[CR8] Castillo UF, Strobel GA, Mullenberg K, Condron MM, Teplow DB, Folgiano V (2006). Munumbicins E-4 and E-5: novel broad-spectrum antibiotics from *Streptomyces* NRRL 3052. FEMS Microbiol Lett.

[CR111] Chen H-H, Qin S, Li J, Zhang Y-Q, Xu L-H, Jiang C-L, Kim C-J, Li W-J (2009) *Pseudonocardia endophytica* sp. nov., isolated from the pharmaceutical plant *Lobelia clavata*. Int J Syst Evolut Microbiol 59:559–563 10.1099/ijs.0.64740-0 19244441

[CR112] Chen M, Zhang L, Zhang X (2011) Isolation and inoculation of endophytic actinomycetes in root nodules of *Elaeagnus angustifolia*. Mod Appl Sci 5(2):p264

[CR9] Chithra S, Jasima B, Sachidanandanb P, Jyothisa M, Radhakrishnana EK (2014). Piperine production by endophytic fungus *Colletotrichum gloeosporioides* isolated from *Piper nigrum*. S Phytomed.

[CR113] Conti R, Cunha IGB, Siqueira VM, Souza-Motta CM, Amorim ELC, Araujo JM (2012) Endophytic microorganisms from leaves of *Spermacoce verticillata* (L.): diversity and antimicrobial activity. J Appl Pharm Sci 2(12):17–22

[CR11] Crawford DL, Lynch JM, Whipps JM, Ousley MA (1993). Isolation and characterization of actinomycete antagonists of a fungal root pathogen. Appl Environ Microbiol.

[CR12] De Bary A (1866). Morphologie und Physiologie der Pilze, Flechten, und Myxomyceten. Hofmeister’s handbook of physiological Botany.

[CR13] Dochhil H, Dkhar MS, Barman D (2013). Seed germination enhancing activity of endophytic *Streptomyces* isolated from indigenous ethno-medicinal plant *Centella asiatica*. Int J Pharm Biol Sci.

[CR14] Doroghazi JR, Metcalf WW (2013). Comparative genomics of actinomycetes with a focus on natural product biosynthetic genes. BMC Genomic.

[CR15] Du H, Su J, Yu L, Zhang Y (2013). Isolation and physiological characteristics of endophytic actinobacteria from medicinal plants. Wei Sheng Wu Xue Bao.

[CR16] Du HJ, Zhang YQ, Liu HY, Su J, Wei YZ, Ma BP (2013). *Allonocardiopsis opalescens* gen. nov., sp. nov., a new member of the suborder *Streptosporangineae*, from the surface-sterilized fruit of a medicinal plant. Int J Syst Evol Microbiol.

[CR114] El-Gendy MMA, EL-Bondkly AMA (2010) Production and genetic improvement of a novel antimycotic agent, saadamycin, against dermatophytes and other clinical fungi from endophytic *Streptomyces* sp. Hedaya48. J Ind Microbiol Biotechnol 37:831–841 10.1007/s10295-010-0729-2 20458610

[CR17] El-Shatoury S, Abdulla H, El-Karaaly O, El-Kazzaz W, Dewedar A (2006). Bioactivities of endophytic actinomycetes from selected medicinal plants in the world heritage site of Saint Katherine. Egypt Int J Bot.

[CR18] El-Shatoury S, El-Kraly O, El-Kazzaz W, Dewedar A (2009). Antimicrobial activities of Actinomycetes inhabiting *Achillea fragrantissima* (Family: *Compositae*). Egypt J Nat Toxins.

[CR19] El-Shatoury SA, El-Kraly OA, Trujillo ME, El-Kazzaz WM, El-Din E-SG, Dewedar A (2013). Generic and functional diversity in endophytic actinomycetes from wild *Compositae* plant species at South Sinai—Egypt. Res Microbiol.

[CR20] El-Tarabily KA (2003). An endophytic chitinase-producing isolate of *Actinoplanes missouriensis*, with potential for biological control of root rot of lupine caused by *Plectosporium tabacinum*. Aust J Bot.

[CR21] El-Tarabily KA, Sivasithamparam K (2006). Nonstreptomycete actinomycetes as biocontrol agents of soil-borne fungal plant pathogens and as plant growth promoters. Soil Biol Biochem.

[CR22] El-Tarabily KA, Hardy GEStJ, Sivasithamparam K (2010). Performance of three endophytic actinomycetes in relation to plant growth promotion and biological control of *Pythium aphanidermatum*, a pathogen of cucumber under commercial field production conditions in the United Arab Emirates. Eur J Plant Pathol.

[CR23] Eyberger AL, Dondapati R, Porter JR (2006). Endophyte fungal isolates from *Podophyllum peltatum* produce podophyllotoxin. J Nat Prod.

[CR115] Ezra D, Castillo UF, Strobel GA, Hess WM, Porter H, Jensen JB, Condron MAM, Teplow DB, Sears J, Maranta M, Hunter M, Weber B, Yaver D (2004) Coronamycins, peptide antibiotics produced by a verticillate *Streptomyces* sp. (MSU-2110) endophytic on *Monstera* sp. Microbiology 150:785–793 10.1099/mic.0.26645-0 15073289

[CR24] Fiedler H, Bruntner C, Riedlinger J, Bull AT, Knutsen G, Goodfellow M (2008). Proximicin A, B and C, novel aminofuran antibiotic and anticancer compounds isolated from marine strains of the actinomycetes *Verrucosispora*. J Antibiot.

[CR25] Gaiero JR, McCall CA, Thompson KA, Day NJ, Best AS, Dunfield KE (2013). inside the root microbiome: bacterial root endophytes and plant growth promotion. Am J Bot.

[CR116] Gangwar M, Dogra S, Sharma N (2011) Antagonistic bioactivity of endophytic actinomycetes isolated from medicinal plants. J Advanced Lab Res Biol 2(4):154–157

[CR26] Gangwar M, Dogra S, Gupta UP, Kharwar RN (2014). Diversity and biopotential of endophytic actinomycetes from three medicinal plants in India. African J Microbiol Res.

[CR27] Goodfellow M, Fiedler HP (2010). A guide to successful bioprospecting: informed by actinobacterial systematics. Ant van Leeuw.

[CR28] Goodfellow M, Kämpfer P, Busse H-J, Trujillo ME, Suzuki K-I, Ludwig W, Whitman WB (2012) Bergey’s manual of systematic bacteriology, 2nd edn, vol 5 The *Actinobacteria*, Part A, Springer, New York

[CR29] Hallmann J, Jeger MJ, Spence NJ (2001). Plant interactions with endophytic bacteria. Biotic interactions in plant-pathogen association.

[CR30] Hallmann J, Berg G, Schulz B, Schulz B, Boyle C, Sieber TN (2006). Isolation procedures for endophytic microorganisms. Soil biology.

[CR31] Hasegawa T, Lechevalier MP, Lechevalier HA (1978). New genus of the *Actinomycetales*: *Actinosynnema* gen. nov. Int J Syst Bacteriol.

[CR32] Hasegawa S, Meguro A, Shimizu M, Nishimura T, Kunoh H (2006). Endophytic actinomycetes and their interactions with host plants. Actinomycetologica.

[CR33] Hayakawa M, Nonomura H (1987). Efficacy of artificial humic acid is a selective nutrient in HV agar used for the isolation of actinomycetes. J Ferment Technol.

[CR34] Hou BC, Wang ET, Li Y, Jia RZ, Chen WF, Man CX (2009). Rhizobial resource associated with epidemic legumes in Tibet. Microb Ecol.

[CR35] Igarashi Y (2004). Screening of novel bioactive compounds from plant-associated actinomycetes. Actinomycetolog.

[CR36] Igarashi Y, Iida T, Sasaki T, Saito N, Yoshida R, Furumai T (2002). Isolation of actinomycetes from live plants and evaluation of antiphytopathogenic activity of their metabolites. Actinomycetolog.

[CR37] Igarashi Y, Miura S, Fujita T, Furumai T (2006). Pterocidin, a cytotoxic compound from the endophytic *Streptomyces hygroscopicus*. J Antibiot.

[CR38] Igarashi Y, Trujillo ME, Martinez-Molina E, Yanase S, Miyanaga S, Obata T (2007). Antitumor anthraquinones from an endophytic actinomycete *Micromonospora lupini* sp. nov. Bioorg Med Chem Lett.

[CR39] Igarashi Y, Mogi T, Yanase S, Miyanaga S, Fujita T, Sakurai H (2009). Brartemicin, an inhibitor of tumor cell invasion from the actinomycete *Nonomuraea* sp. J Nat Prod.

[CR40] Ikeda H, Ishikawa J, Hanamoto A, Shinose H, Kikuchi T, Shiba Y (2003). Complete genome sequence and comparative analysis of the industrial microorganism *Streptomyces avermitilis*. Nat Biotechnol.

[CR41] Ivantiskaya LP, Singal SM, Bibikova MV, Vostrov SN (1978). Direct isolation of Micromonospora on selective media with gentamicin. Antibiot.

[CR42] Janso JE, Carter GT (2010). Biosynthetic potential of phylogenetically unique endophytic actinomycetes from tropical plants. Appl Environ Microbiol.

[CR117] Kafur A, Khan AB (2011) Isolation of endophytic actinomycetes from *Catharanthes roseus* (L.) G. Don leaves and their antimicrobial activity. Iranian J Biotechnol 9(4):301–306

[CR43] Kim N, Shin JC, KimW Hwang BY, Kim BS, Hong YS (2006). Cytotoxic 6-alkylsalicylic acids from the endophytic *Streptomyces laceyi*. J Antibiot.

[CR118] Kumar U, Singh A, SivaKumar T (2011) Isolation and screening of endophytic actinomycetes from different parts of *Emblica officinalis*. Ann Biol Res 2(4):423–434

[CR44] Kumar A, Patil D, Rajamohanan PR, Ahmad A (2013). Isolation, purification and characterization of from endophytic fungus *Fusarium oxysporum* isolated from *Catharanthus roseus*. PLoS ONE.

[CR45] Küster E (1959). Outline of a comparative study of criteria used in characterization of the actinomycetes. Int Bull Bacteriol Nomencl Taxon.

[CR46] Küster E, Williams ST (1964). Selection of media for isolation of* Streptomycetes*. Nature.

[CR47] Li J, Zhao GZ, Chen HH, Qin S, Xu LH, Jiang CL (2008). *Rhodococcus cercidiphylli* sp. nov., a new endophytic actinobacterium isolated from a *Cercidiphyllum japonicum* leaf. Syst Appl Microbiol.

[CR119] Lin ZJ, Lu XM, Zhu TJ, Fang YC, Gu QQ, Zhu W (2008) GPR12 selections of the metabolites from an endophytic *Streptomyces* sp. asociated with *Cistanches deserticola*. Arch Pharm Res 31(9):1108–1114 10.1007/s12272-001-1276-4 18806952

[CR48] Lu C, Shen Y (2003). A new macrolide antibiotic with antitumor activity produced by *Streptomyces* sp. CS, a commensal microbe of *Maytenus hookeri*. J Antibiot.

[CR49] Lu C, Shen Y (2007). A novel ansamycin, naphthomycin k from *Streptomyces* sp. J Antibiot.

[CR50] Ludwig W, Klenk HP (2005) Overview: a phylogenetic backbone and taxonomic framework for prokaryotic systematics. In: Brenner DJ, Krieg NR, Staley JT, Garrity GM (eds) Bergey’s manual of systematic bacteriology, 2nd edn, vol 2, the *Proteobacteria*, part A, introductory essays. Springer, New York pp 49–65

[CR51] Machavariani NG, Ivankova TD, Sineva ON, Terekhova LP (2014). Isolation of endophytic actinomycetes from medicinal plants of the Moscow region, Russia. World Appl Sci J.

[CR52] Minamiyama H, Shimizu M, Kunoh H, Furumai T, Igarashi Y, Onaka H (2003). Multiplication of isolate R-5 of *Streptomyces galbus* on rhododendron leaves and its production of cell wall-degrading enzymes. J Gen Plant Pathol.

[CR53] Mincer TJ, Jensen PR, Kauffman CA, Fenical W (2002). Widespread and persistent populations of a major new marine actinomycete taxon in ocean sediments. Appl Environ Microbiol.

[CR120] Mini Priya R (2012) Endophytic actinomycetes from Indian medicinal plants as antagonists to some phytopathogenic fungi. Open Access Scient Rep 1(4):259

[CR54] Mitchell AM, Strobel GA, Moore E, Robison R, Sears J (2010). Volatile antimicrobials from *Muscodor crispans*, a novel endophytic fungus. Microbiology.

[CR121] Moussa HE, El-Shatoury SA, Wahid OAA, Dewedar A (2011) Characterization of endophytic actinomycetes from wild Egyptian plants as antagonists to some phytopathogenic fungi. Egyptian J Nat Toxins 8(1, 2):32–48

[CR55] Newman DJ, Cragg GM (2007). Natural products as sources of new drugs over the last 25 years. J Nat Prod.

[CR56] Nimnoi P, Pongsilp N, Lumyong S (2010). Genetic diversity and community of endophytic actinomycetes within the roots of *Aquilaria crassna* Pierre ex Lec assessed by actinomycetes-specific PCR and PCR-DGGE of 16S rRNA gene. Biochem Syst Ecol.

[CR57] Olano C, Méndez C, Salas JA (2009). Antitumor compounds from actinomycetes from gene clusters to new derivatives by combinatorial synthesis. Nat Prod Rep.

[CR58] Oliynyk M, Samborskyy M, Lester JB, Mironenko T, Scott N, Dickens S (2007). Complete genome sequence of the erythromycin-producing bacterium *Saccharopolyspora erythraea* NRRL 23338. Nat Biotechnol.

[CR59] Ōmura S, Ikeda H, Ishikawa J (2001). Genome sequence of an industrial microorganism *Streptomyces avermitilis*: deducing the ability of producing secondary metabolites. Proc Natl Acad Sci.

[CR60] Pandey B, Ghimir P, Agrawal VP (2004) Studies on the antibacterial activity of the actinomycetes isolated from the Khumbu Region of Nepal. International Conference on the Great Himalayas: Climate, Health, Ecology, Management and Conservation, Kathmandu, Organized by Kathmandu University and the Aquatic Ecosystem Health and Management Society, Canada

[CR61] Parrent JL, Peay K, Arnold AE, Comas L, Avis P, Tuininga A (2010). Moving from pattern to process in fungal symbioses: linking functional traits, community ecology, and phylogenetics. New Phytol.

[CR62] Passari AK, Mishra VK, Saikia R, Gupta VK, Singh BP (2015). Isolation, abundance and phylogenetic affiliation of endophytic actinomycetes associated with medicinal plants and screening for their in vitro antimicrobial biosynthetic potential. Front Microbiol.

[CR63] Petrini O, Sieber TN, Toti L, Viret O (1992). Ecology, metabolite production, and substrate utilization in endophytic fungi. Nat Tox.

[CR64] Pujiyanto S, Lestari Y, Suwanto A, Budiarti S, Darusman LK (2012). Alpha-glucosidase inhibitor activity and characterization of endophytic actinomycetes isolated from some Indonesian diabetic medicinal plants. Int J Pharm Pharm Sci.

[CR122] Pullen C, Schmitz P, Meurer K, Bamberg DD, Lohman S, De Castro Franca S, Groth I, Schlegel B, Mollmann U, Gollmick F, Grafe U, Leistner E (2002) New and bioactive compounds from *Streptomyces* strains residing in the wood of *Celastraceae*. Planta 216:162–167 10.1007/s00425-002-0874-6 12430026

[CR65] Qin S, Li J, Zhao GZ, Chen HH, Xu LH, Li WJ (2008). *Saccharopolyspora endophytica* sp. nov., an endophytic actinomycete isolated from the root of *Maytenus austroyunnanensis*. Syst Appl Microbiol.

[CR66] Qin S, Wang HB, Chen HH, Zhang YQ, Jiang CL, Xu LH (2008). *Glycomyces endophyticus* sp. nov., an endophytic actinomycete isolated from the root of *Carex baccans* Nees. Int J Syst Evol Microbiol.

[CR67] Qin S, Jie L, Hua-Hong C, Guo-Zhen Z, Wen-Yong Z, Cheng-Lin J (2009). Isolation diversity and antimicrobial activity of rare actinobacteria from medicinal plants of tropical rain forests in Xishuangbanna. China App Environ Microbiol.

[CR106] Qin S, Xing K, Jiang J, Xu L, Li W (2011) Biodiversity, bioactive natural products and biotechnological potential of plant-associated endophytic actinobacteria. Appl Microbiol Biotechnol 89:457–473 10.1007/s00253-010-2923-6 20941490

[CR107] Qin S, Yuan B, Zhang YJ, Bian GK, Tamura T, Sun BZ, Li WJ, Jiang JH (2012a) *Nocardioides panzhihuaensis* sp. nov., a novel endophytic actinomycete isolated from medicinal plant *Jatropha curcas* L. Ant van Leeuw 102:353–360 10.1007/s10482-012-9745-8 22552630

[CR68] Qin S, Chen HH, Zhao GZ, Li J, Zhu WY, Xu LH (2012). Abundant and diverse endophytic actinobacteria associated with medicinal plant *Maytenus austroyunnanensis* in Xishuangbanna tropical rainforest revealed by culture-dependent and culture-independent methods. Environ Microbiol Rep.

[CR69] Rai M, Agarkar G, Rathod D, Gurib-Fakim A (2014). Multiple applications of endophytic *Colletotrichum* species occurring in medicinal plants, in novel plant bioresources: Applications in food, medicine and cosmetics. Novel plant bioresources.

[CR70] Rai M, Rathod D, Agarkar G, Dar M, Brestic M, Marostica Junior MR (2014). Fungal growth promotor endophytes: a pragmatic approach towards sustainable food and agriculture. Symbiosis.

[CR71] Rosselló-Mora R, Amann R (2001). The species concept for prokaryotes. FEMS Microbiol.

[CR72] Rungin S, Indanand C, Suttiviriya P, Kruasuwan W, Jaemsaeng R, Thamchaipenet A (2012). Plant growth enhancing effects by a siderophore producing endophytic streptomycete isolated from a Thai jasmine rice plant (*Oryza sativa* L. cv. KDML105). Ant van Leeuw.

[CR73] Rya RPK, Germaine A, Franks DJ, Ryan DN (2007). Bacterial endophytes: recent developments and applications. FEMS Microbiol Lett.

[CR75] Schulz B, Boyle C, Sieber TN (2006). Microbial root endophytes. What are endophytes?.

[CR76] Schulz D, Nachtigall J, Riedlinger J, Schneider K, Poralla K, Imhoff JF (2009). Piceamycin and its N-acetylcysteine adduct is produced by *Streptomyces* sp. GB 4-2. J Antibiot.

[CR77] Sharma M (2014). Actinomycetes: source, identification, and their applications. Int J Curr Microbiol Appl Sci.

[CR123] Shenpagam NH, Kanchana D, Sinduja G, Sandhya R (2012) Isolation of endophytic actinomycetes from medicinal plants and its mutational effect in biocontrol activity. Int J Pharm Sci Res 3(11):4338–4344

[CR78] Shimizu M, Maheshwari DK (2011). Endophytic actinomycetes: biocontrol agents and growth promoters. Bacteria in agrobiology: plant growth responses.

[CR79] Shutsrirung A, Chromkaew Y, Pathom-Aree W, Choonluchanon S, Boonkerd N (2014). Diversity of endophytic actinomycetes in mandarin grown in northern Thailand, their phytohormone production potential and plant growth promoting activity. Soil Sci Plant Nutr.

[CR82] Staniek A, Woerdenbag HJ, Kayser O (2008). Endophytes: exploiting biodiversity for the improvement of natural product-based drug discovery. J Plant Interact.

[CR83] Stierle A, Strobel G, Stierle D (1993). Taxol and taxane production by *Taxomyces andreanae*, an endophytic fungus of pacific yew. Science.

[CR84] Strobel G, Daisy B (2003). Bioprospecting for microbial endophytes and their natural products. Microbiol Mol Biol Rev.

[CR124] Sunaryanto R, Mahsunah AH (2013) Isolation, purification, and characterization of antimicrobial substances from endophytic actinomycetes. Makara J Sci 17(3):87–92

[CR85] Taechowisan T, Lumyong S (2003). Activity of endophytic actinomycetes from roots of *Zingiber officinale* and *Alpinia galanga* against phytopathogenic fungi. Ann Microbiol.

[CR86] Taechowisan T, Peberdy JF, Lumyong S (2003). Isolation of endophytic actinomycetes from selected plants and their antifungal activity. World J Microbiol Biotechnol.

[CR87] Taechowisan T, Lu C, Shen Y, Lumyong S (2005). Secondary metabolites from endophytic *Streptomyces aureofaciens* CMUAc130 and their antifungal activity. Microbiology.

[CR88] Taechowisan T, Wanbanjob A, Tuntiwachwuttikul P, Taylor WC (2006). Identification of *Streptomyces* sp. Tc022, an endophyte in *Alpinia galanga*, and the isolation of actinomycin D. Ann Microbiol.

[CR89] Taechowisan T, Lu C, Shen Y, Lumyong S (2007). Antitumor activity of 4-arylcoumarins from endophytic *Streptomyces**aureofaciens* CMUAc130. J Cancer Res Ther.

[CR90] Takahashi Y, Omura S (2003). Isolation of new actinomycete strains for the screening of new bioactive compounds. J Gen Appl Microbiol.

[CR91] Tan HM, Cao LX, He ZF, Su GJ, Lin B, Zhou SN (2006). Isolation of endophytic actinomycetes from different cultivars of tomato and their activities against *Ralstonia solanacearum* in vitro. World J Microbiol Biotechnol.

[CR92] Tanvir R, Sajid I, Hasnain S (2014). Larvicidal potential of *Asteraceae* family endophytic actinomycetes against *Culex quinquefasciatus* mosquito larvae. Nat Prod Res.

[CR93] Udwary DW, Ziegler L, Asolbar HN, Singan V, Lapidus A, Fenical W (2007). Genome sequencing reveals complete secondary metabolome in the marine actinomycete *Salinispora tropica*. Proc Natl Acad Sci USA.

[CR94] Verma VC, Gond SK, Kumar A, Mishra A, Kharwar RN, Gnage A (2009). Endophytic actinomycetes from *Azadirachta indica* A. Juss.: isolation, diversity, and anti-microbial activity. Microbial Ecol.

[CR95] Wang HX, Geng ZL, Zeng Y, Shen YM (2008). Enrichment plant microbiota for a metagenomic libraryconstruction. Environ Microbiol.

[CR96] Wang P, Kong F, Wei J, Wang Y, Wang W, Hong K (2014). Alkaloids from the mangrove-derived actinomycete *Jishengella endophytica* 161111. Mar Drug.

[CR97] Wayne LG, Brenner DJ, Colwell RR, Grimont PAD, Kandler O, Krichevsky ML (1987). Report of the *ad hoc* committee on reconciliation of approaches to bacterial systematics. Int J Syst Bacteriol.

[CR98] Williams ST, Davies FL (1965). Use of antibiotics for selective isolation and enumeration of actinomycetes in soil. J Gen Microbiol.

[CR100] Zhang X, Ren K, Zhang L (2012). Screening and preliminary identification of medicinal plants endophytic actinomycetes used for inhibiting penicillin-resistant *Staphylococcus**aureus*. Int J Biol.

[CR101] Zhang YJ, Zhang WD, Qin S, Bian GK, Xing K, Li YF (2013). *Saccharopolyspora dendranthemae* sp. nov. a halotolerant endophytic actinomycete isolated from a coastal salt marsh plant in Jiangsu, China. Ant van Leeuw.

[CR102] Zhang J, Wang JD, Liu CX, Yuan JH, Wang XJ, Xiang WS (2014). A new prenylated indole derivative from endophytic actinobacteria *Streptomyces* sp. neau-D50. Nat Prod Res.

[CR103] Zhao K, Penttinen P, Guan T, Xiao J, Chen Q, Xu J (2011). The diversity and anti-microbial activity of endophytic actinomycetes isolated from medicinal plants in Panxi Plateau China. Curr Microbiol.

[CR104] Zhou H, Yang Y, Zhang J, Peng T, Zhao L, Xu L (2013). Alkaloids from an endophytic *Streptomyces* sp. YIM66017. Nat Prod Commun.

[CR105] Zhou H, Yang Y, Peng T, Li W, Zhao L, Xu L, Ding Z (2014). Metabolites of *Streptomyces* sp., an endophytic actinomycete from *Alpinia oxyphylla*. Nat Prod Res.

